# Rapidly developed heterotopic ossification: a rare complication after trauma

**DOI:** 10.1093/jscr/rjac086

**Published:** 2022-04-18

**Authors:** Jae H T Lee, Janaka Balasooriya, Thembekile Ncube

**Affiliations:** Department of General Surgery, The Canberra Hospital, Garran, Australian Capital Territory, Australia; Department of General Surgery, The Canberra Hospital, Garran, Australian Capital Territory, Australia; Department of General Surgery, The Canberra Hospital, Garran, Australian Capital Territory, Australia

## Abstract

Heterotopic ossification (HO) is a condition where aberrant bone grows in tissues. This case study presents a rare complication of trauma and laparotomies, where the rapid and extensive occurrence of HO has delayed abdominal incision closure resulting in multiple surgeries and prolonged recovery. A 44-year-old man was retrieved after a truck accident resulting in multi-organ injuries. He required damage control trauma laparotomy followed by several relooks and multiple orthopaedic procedures. Despite several attempts, approximation of the laparostomy wound was not possible due to abdominal rigidity. Computed tomography scans done 20 days after injury demonstrated advanced HO over the wound edge. Early development of HO may explain why the abdominal incision was difficult to close and highlights the importance of being aware of HO as an early complication after trauma and midline laparotomy.

## INTRODUCTION

Heterotopic ossification (HO) is a condition, in which ectopic bone is formed in tissues where it does not belong [1, 2]. Traumatic HO generally occurs in a delayed manner, 4–12 weeks after the injury, predominantly in males (89%), and has been reported in wounds, kidneys, mesentery, gastrointestinal tract and abdominal incisions [2–6]. Few case studies describe HO as a complication after midline abdominal incisions, but this generally occurs months after surgery [6–9]. HO clinically manifests with non-specific symptoms of fever, warmth and erythema around the affected sites causing loss of function [2]. Given the nonspecific nature of HO, differentials are wide. The diagnosis is difficult, but history of trauma and imaging can confirm ectopic bony growth. The pathogenesis of HO is unclear. It is theorized to involve either seeding of osteogenic cells or stimulation and transformation of multipotent mesenchymal cells present in the connective tissue septa within muscle, into osteogenic cells by bone morphogenic protein [2, 5, 8].

Here we describe a rare case of a rapidly growing and extensive anterior abdominal wall HO that prohibited the closure of a midline incision after a trauma laparotomy and multiple relooks. This complication resulted in increased morbidities and utilization of an unconventional method to close the abdominal incision months later.

## CASE REPORT

Mr. R, 44-year-old male, was a driver in a high-speed collision of two trucks with cabin intrusions and prolonged extrication. His medical and surgical history included psoriatic arthritis, gastro-oesophageal reflux disease, hiatus hernia repair and a Roux-en-Y gastric bypass for weight reduction.

Mr R. sustained multiple traumatic abdominal and orthopaedic injuries and was haemodynamically compromised with a systolic blood pressure of 60 mmHg, haemoglobin of 52 g/L at arrival, despite 6 units of packed red blood cell transfusions during the retrieval. Damage control trauma laparotomy found to have two bleeding small bowel mesenteric tears, de-vascularized ileum and caecal perforation resulting in 40 cm of ileum and ileocaecal resection. After surgery, the patient was transferred to ICU with a temporary abdominal closure. Computed tomography (CT, pan scan) and X-rays were performed post-surgery and revealed small bilateral pneumothoraces, left 8th and 10th rib fractures and multiple long bone fractures including right wrist distal radius fractures (Table 1).

**Table 1:** Injury list

**Anatomical locations**	**Injuries**
Chest	Bilateral pneumothoraces
	Left 8th and 10th rib fractures
Small bowel	Hemoperitonium, large small bowel mesentery tears, multiple
	Microperforation of small bowel
Caecum	Large mesentery tears, serosal tear, multiple microperforation
Humerus	Right open midshaft fracture
Wrist	Right distal radius fracture
Femur	Right femoral midshaft transverse fracture
Tibia	Right tibia shaft fracture
Foot	Right talar neck fracture/pilon fracture
Fibula	Right midshaft and distal fibula fracture

Two days after the initial trauma laparostomy, the proximal end of the small bowel was anastomosed with the remaining terminal ileum, and an ileostomy was fashioned. Multiple large bowel contusions were further noted and the abdominal wall was closed. Unfortunately, 4 days later, the end ileostomy became ischemic prompting a return to operating theatre to resect the ileostomy and remaining 35 cm of terminal ileum that had become non-viable. The proximal small bowel end was stapled and a temporary abdominal closure was performed. Another relook was done at Day 5 to create a new ileostomy, but due to the tension of the abdominal wall muscle, the anterior abdominal wound closure was not achievable. Over the following 18 days, Mr. R underwent multiple orthopaedic surgeries and abdominal wound approximation was re-attempted without success.

**Figure 1:**
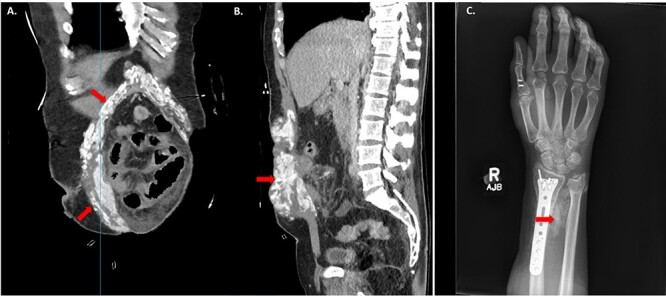
HO demonstrated on computed tomography (CT) imaging and X-ray after traumatic Injuries. (**A**) Coronal image of HO and (**B**) Sagittal image of HO on CT abdomen and pelvis demonstrating sites of HO (red arrows). (**C**) X-ray of right forearm between ulna and radius showing HO (red arrows).

Approximately 20 days from the initial accident, the patient developed persistent fever, tachycardia, tachypnoea and swelling on the right wrist, by this time he was not able to flex forward and had limitation to his wrist movement. CT scan of the abdomen revealed advanced calcified HO surrounding the midline incision (Fig. 1A and B). In addition, heavily calcified HO to the right wrist triangular fibrocartilage between the ulnar and radius was demonstrated on ultrasound and X-ray (Fig. 1C). In conjunction with the development of his symptoms, the calcium levels increased to the range of 2.77 mmol/L to 2.87 mmol/L for 3 weeks and then returned to normal range.

**Figure 2:**
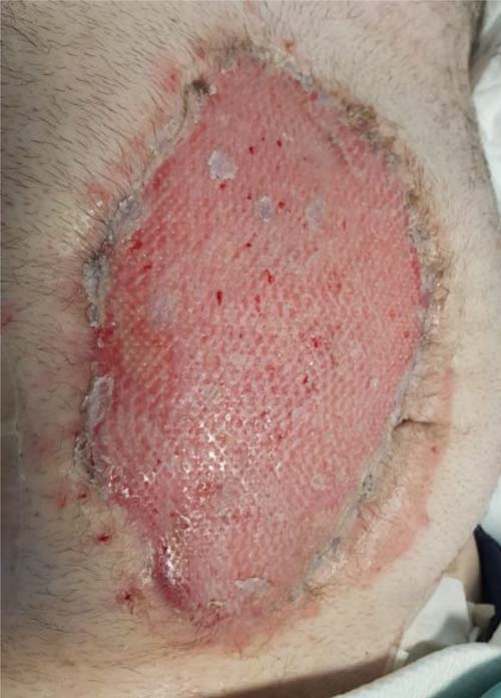
Split thickness skin graft for abdominal incision closure.

Three months later, repeated applications of vacuum dressings promoted sufficient granulation over the open abdominal wound allowing an autologous split thickness skin graft to achieve abdominal closure (Fig. 2). A couple of weeks after the closure, the patient commenced rehabilitation and subsequently discharge home. HO of the abdomen and wrist was surgically excised at a later date and the right wrist was followed by prophylactic radiotherapy.

Since then, the patient has recovered fully.

## DISCUSSION

In this case study, we described a rare complication of extensive and rapidly developed abdominal and wrist HO after trauma. To date, there has not been a case reported in literature, where HO developed earlier than 20 days. Although advanced HO was detected about 20 days, subclinical HO would have started to develop just days after trauma and surgeries. The nature of the injuries and the number of surgeries may have contributed to early development of HO. This subclinical HO may have contributed to initial failed attempts of abdominal wound closure. HO can be visualized on plain film X-ray, bone scans, CT or magnetic resonance imaging findings (MRI) [1, 8]. For soft tissue ossification, CT can detect HO in early stages. HO as a post-operative complication after closure of midline incisions has been reported and described based on CT and MRI [2, 3, 5]. The case studies that demonstrate abdominal HO are after a complex ventral hernia repair, abdominal aortic aneurysm repair, achalasia, gastric reduction procedure for obesity and total colectomy [1, 5, 10–12].

There is no standardized treatment for HO. Asymptomatic HO is often managed conservatively, but symptomatic HO causing pain and discomfort can be managed prophylactically with non-steroidal anti-inflammatory drugs, localized low dose radiation or combined treatments [2, 8, 13]. To address complications of HO such as loss of function and bowel perforation internally, the only effective treatment for formed HO is delayed (12–18 months) excision. Reoccurrence rate is high as additional iatrogenic trauma to the surrounding tissues can again promote HO [2].

In this case report, we presented a rare complication after abdominal trauma. The patient developed early and extensive HO around the abdominal midline incision site, which made the closure of the anterior abdominal wall possible only through utilizing of skin grafts. The current case recognizes HO as a rare early complication after trauma and abdominal incision. It is an important problem to be aware to reduce morbidity after traumatic injuries.
